# A Hospital Letters Exchange Bureau

**Published:** 1912-11-23

**Authors:** 


*To the Editor of* The Hospital.

### A Hospital Letters Exchange Bureau.

*To the Editor of* The Hospital.

Sir,—All institutions where the letter system is in vogue have, I believe, found a difficulty in obtaining the necessary letters from patients who apply for treatment, and are compelled either to refuse advice, which is contrary to their wish and desire, or to treat the individual without letter, which is contrary to their rule, and not only the institution but the parochial clergyman and the philanthropic worker finds a great difficulty in regard to letters; he may have dozens of one particular kind of letter sent him for distribution in the course of a year, for which he never has an applicant, and hence all the letters are wasted; whereas, on the other hand, he receives applications for letters for other institutions, and is totally ignorant whence to obtain them; it means in many cases dozens of written applications to subscribers found in the hospital reports.

Would it not be possible to have a "Hospital Letters Exchange Bureau," where clergy, heads of societies, or other responsible persons might exchange letters for which they have no need with those who require them and *vice versâ*? Thus we feel the worthy poor would be materially benefited and the financial machinery of our hospitals made to run smoothly.—Yours faithfully, B. R.

[This suggestion is not a new one, but the various attempts made to put it into practice have never been very successful.—Ed. The Hospital.]

